# Haemoglobin concentrations in pregnancy and respiratory and allergic outcomes in childhood: Birth cohort study

**DOI:** 10.1111/cea.13034

**Published:** 2017-10-16

**Authors:** S. O. Shaheen, C. Macdonald‐Wallis, D. A. Lawlor, A. J. Henderson

**Affiliations:** ^1^ Centre for Primary Care and Public Health Barts and The London School of Medicine and Dentistry London UK; ^2^ School of Social and Community Medicine University of Bristol Bristol UK; ^3^ MRC Integrative Epidemiology Unit at the University of Bristol Bristol UK

**Keywords:** allergic sensitisation, ALSPAC, anaemia, birth cohort, haemoglobin

## Abstract

**Background:**

Limited epidemiological evidence suggests that low maternal iron status and anaemia in pregnancy may increase the risk of childhood respiratory and allergic outcomes.

**Objectives:**

To investigate the relation between maternal haemoglobin concentrations in pregnancy and childhood respiratory and allergic outcomes.

**Methods:**

In the Avon Longitudinal Study of Parents and Children (ALSPAC), we examined associations of maternal haemoglobin concentrations (g/dL) in pregnancy with hayfever, eczema, wheezing, doctor‐diagnosed asthma, allergic sensitisation and total IgE at 7 years, and with lung function at 8‐9 years in the offspring, after controlling for potential confounders (N = 3234‐5335).

**Results:**

Maternal haemoglobin was not associated with offspring hayfever, eczema, wheezing or asthma. However, the first haemoglobin measurement in pregnancy (<18 weeks' gestation) and the last measurement (>28 weeks' gestation) were negatively associated with allergic sensitisation (adjusted odds ratio [95% CI] per g/dL 0.91 [0.83 to 0.99] and 0.90 [0.83 to 0.98], respectively). The last haemoglobin measurement was also negatively associated with total IgE (adjusted geometric mean ratio 0.94 [0.88 to 0.99]). Anaemia (haemoglobin <11 g/dL) in late pregnancy was negatively associated with forced vital capacity (difference in standard deviation score −0.07 [−0.13 to −0.01]).

**Conclusions and Clinical Relevance:**

Lower maternal haemoglobin in pregnancy may be a risk factor for allergic sensitisation, elevated IgE and lower FVC in childhood, which may reflect effects of lower prenatal iron status. However, maternal haemoglobin was not associated with risk of childhood asthma or other allergic disorders.

## 1 BACKGROUND

There is a substantial body of epidemiological evidence implicating the prenatal environment in the aetiology of childhood respiratory and allergic disorders,^1^ and there has been considerable interest in the possible role of maternal nutrition during pregnancy.^2^ One potentially modifiable risk factor which has received relatively little attention is iron deficiency, which is common in pregnant women in the West.^3^ Prenatal iron deficiency could plausibly influence respiratory and allergic outcomes by causing prematurity and impaired foetal growth; gestational age at delivery and offspring birthweight are associated with maternal haemoglobin concentration,^4^ as well as with offspring wheezing, asthma, eczema, allergic sensitisation and impaired lung function.^5^, ^6^, ^7^, ^8^ Also, animal data suggest that an adequate supply of iron is required for optimal lung, and particularly airway, development.^9^

The first clues that prenatal iron status might be important came from the Avon Longitudinal Study of Parents and Children (ALSPAC), the study used in this paper, in which we found that umbilical cord iron concentration in over 2000 participants was negatively associated with wheezing and eczema in early childhood.^10^ More recently, another UK birth cohort study (Study of Eczema and Asthma to Observe the influence of Nutrition, SEATON) reported results of exploratory analyses in a small subgroup (n = 157) of that cohort. The authors found some evidence to suggest that different indicators of lower maternal iron status in early gestation were associated with an increased risk of wheezing and lower lung function in the offspring at 10 years of age, although findings were inconsistent across the different indicators, and no associations were found with asthma or eczema.^11^ A small study from the USA (n = 97) suggested that poor foetal iron status may increase the risk of infant eosinophilia.^12^

Iron deficiency is the commonest cause of anaemia in pregnancy.^13^, ^14^ A study from the USA (N = 597) specifically investigated the relation between anaemia in pregnancy and respiratory outcomes in childhood, and found that maternal anaemia was positively associated with risk of early childhood wheezing; positive associations were also found with persistent wheezing and asthma at 6 years of age, but only amongst offspring of mothers with asthma.^15^ On the other hand, a prospective study conducted in the Netherlands did not confirm any association between maternal haemoglobin levels during pregnancy and wheezing in early childhood or asthma outcomes at the age of 6 years.^16^

In the population‐based ALSPAC birth cohort, we have investigated whether lower maternal haemoglobin concentrations in pregnancy, as a proxy for low prenatal iron status, are associated with an increased risk of respiratory and allergic outcomes in childhood.

## 2 METHODS

### 2.1 Participants

The Avon Longitudinal Study of Parents and Children (ALSPAC) is a population‐based birth cohort that recruited 14 541 predominantly white pregnant women resident in Avon, UK with expected dates of delivery 1st April 1991 to 31st December 1992. Of these pregnancies, there were 14 676 fetuses, resulting in 14 062 live births and 13 978 singleton or twin children who were alive at 1 year of age. Of these, 13 758 had maternal obstetric information abstracted from medical records. The cohort has been followed since birth with annual questionnaires and, since age 7 years, with objective measures in annual research clinics. The study protocol has been described previously,^17^, ^18^ and further information can be found at: http://www.alspac.bris.ac.uk. Please note that the study website contains details of all the data that are available through a fully searchable data dictionary: http://www.bris.ac.uk/alspac/researchers/data-access/data-dictionary/. Ethics approval for all aspects of data collection was obtained from the ALSPAC Ethics and Law Committee (IRB 00003312) and the Local NHS Research Ethics Committees. Written consent was obtained for clinic measurements.

### 2.2 Maternal haemoglobin in pregnancy

All blood haemoglobin measurements which were taken as part of routine antenatal care were abstracted from the women's obstetric records by six trained research midwives. There was no between‐midwife variation in mean values of the data abstracted and error rates were consistently <1% in repeated data entry checks. There was a median of 3 measurements of haemoglobin per woman (interquartile range of 2 to 3). Gestational age at each measurement was calculated from the date of measurement and the final clinical estimate of the expected date of delivery abstracted from obstetric records. We derived the first haemoglobin as the haemoglobin measurement with the earliest gestational age for each woman, provided that this was before 18 weeks of gestation. If there was not a measurement prior to 18 weeks of gestation, this variable was treated as missing. We derived the last haemoglobin as the haemoglobin measurement with the latest gestational age, provided that this was after 28 weeks of gestation, otherwise this variable was set to missing. Anaemia after 28 weeks of gestation was defined according to WHO criteria, namely a haemoglobin concentration <11 g/dL.^14^ Few women were anaemic by this definition before 18 weeks of gestation.

### 2.3 Outcomes

When the children were 7.5 years old, mothers were asked: “Has your child had any of the following in the past 12 months: wheezing; asthma; eczema; hayfever?.” Children were defined as having current doctor‐diagnosed asthma at 7.5 years if mothers responded positively to the question “Has a doctor ever actually said that your study child has asthma?” and positively to one or both of the questions on wheezing and asthma in the past 12 months.

Allergic sensitisation at 7 years was defined as a positive reaction (any detectable weal) to *D. pteronyssinus*, cat or grass (after subtracting positive saline reactions from histamine and allergen weals, and excluding children unreactive to 1% histamine). (Allergic sensitisation defined in this way identified 96% of children sensitized to 26 other allergens in this cohort^19^). Serum total IgE (kU/L) was measured by fluoroimmunoassay using the Pharmacia UNICAP system (Pharmacia & Upjohn Diagnostics AB, Uppsala, Sweden).

Lung function was measured by spirometry (Vitalograph 2120) at age 8½ years after withholding short‐acting bronchodilators for at least 6 hours and long‐acting bronchodilators and theophyllines for at least 24 hours. The best of three reproducible flow‐volume curves was used to measure FEV_1_, FVC and maximal mid‐expiratory flow (FEF_25‐75_). Lung function measurements were transformed to age, height and gender‐adjusted standard deviation units.^20^ The tests adhered to American Thoracic Society (ATS) criteria for standardisation and reproducibility of flow‐volume measurement,^21^ with the exception of ATS recommendations for duration of expiration^22^; as many children did not fulfil forced expiratory time >6 seconds end of test criteria, a minimal volume change over the final 1 second was used.

### 2.4 Maternal covariates

Maternal age at delivery was abstracted from obstetric records. Information on maternal asthma, eczema, pre‐pregnancy weight and height, parity, smoking status, highest educational qualification and ethnicity was obtained from questionnaires administered during pregnancy. Pre‐pregnancy body mass index (BMI) was calculated as weight(kg)/height(m)^2^ and classed according to World Health Organisation definitions of underweight (<18.5 kg/m^2^), normal (18.5‐24.9 kg/m^2^), overweight (25.0‐29.9 kg/m^2^) and obese (≥30.0 kg/m^2^). Smoking status was categorised as: “Never” for women who did not smoke regularly immediately prior to or during pregnancy; “pre‐pregnancy/first trimester” for women who smoked immediately prior to pregnancy or in the first 3 months and then stopped; and “throughout” for women who continued to smoke after the first 3 months. Maternal highest educational qualification was categorised as Certificate of Secondary Education (CSE)/vocational (exams usually taken when the participants were aged 16 years, the legal minimal age that children could leave school at that time), O level (ordinary‐level; also taken at 16 but more academically challenging than CSE), A level (advanced‐level; taken at age 18 for those staying on at school and required to enter higher education and for some professional occupations), or university degree. Maternal ethnicity was categorised as white or non‐white.

### 2.5 Statistical analysis

We compared the distributions of child and maternal variables across quintiles of first and last maternal haemoglobin in pregnancy, as well as between those who had complete data on all exposure, outcome and co‐variables to be included in the analysis and those excluded because of missing data, using one‐way analysis of variance to test for differences in the means of continuous variables and chi‐squared tests for differences in the categorical variables.

We used logistic regression to relate maternal haemoglobin to binary offspring outcomes: hayfever, eczema, wheezing, asthma and allergic sensitisation, and we present odds ratios for these outcomes. Linear regression was used to relate maternal haemoglobin to continuous offspring outcomes: FEV_1_, FVC and FEF_25‐75_ SD scores and IgE. FEV_1_, FVC and FEF_25‐75_ SD scores were normally distributed and we present mean differences in these outcomes for different levels of maternal haemoglobin (with null value 0), while IgE was natural log‐transformed and we present ratios of geometric means for this outcome. We fitted separate regression models with maternal first or last haemoglobin as the exposure. We analysed maternal haemoglobin as a categorical exposure, using quintiles of haemoglobin, to allow for a non‐linear pattern of association (setting the middle quintile as the reference category, given reports that maternal haemoglobin has a U‐shape association with other outcomes such as low birth weight and pre‐term birth^4^), and separately as a continuous exposure to test for trend. Secondarily, we analysed anaemia after 28 weeks of gestation as a binary exposure.

For all regression analyses, three stages of adjustment were used. In Model 1, we adjusted for the gestational age at the haemoglobin measurement and offspring sex only. In Model 2, we adjusted additionally for potential maternal confounders, namely maternal asthma, eczema, pre‐pregnancy BMI, age, parity, smoking during pregnancy, highest educational qualification, twin pregnancy and ethnicity. In Model 3, we adjusted additionally for gestational age at delivery and offspring birthweight, which are associated with maternal haemoglobin concentration,^4^ as well as with offspring wheezing, asthma, eczema, allergic sensitisation and impaired lung function,^5^, ^6^, ^7^, ^8^ and could mediate the relation between maternal haemoglobin and childhood outcomes. We have made some key assumptions which are necessary for mediation analysis, namely that there is no interaction between the exposures and mediators^23^ and no unmeasured confounding of the mediator‐outcome associations.^24^ Online supplement Figure S1 shows a directed acyclic graph to illustrate potential confounders and mediators of the associations between maternal haemoglobin and childhood outcomes.

In view of previous observations,^15^ we tested whether the associations between maternal haemoglobin and each of the outcomes were modified by maternal asthma, by testing for an interaction term using likelihood ratio tests. All statistical analyses were completed using Stata version 13.1 (StataCorp LP, USA).

## 3 RESULTS

Figure S2 shows the participant flow in the ALSPAC birth cohort, and numbers of mother/child pairs with complete data according to childhood outcomes. Characteristics of mothers and offspring who were included in the analyses of each childhood outcome and who were excluded from all analyses because of incomplete data are shown in supplementary Table S1. Mothers with incomplete data were more likely to be smokers, less well educated, multiparous, younger and non‐white, than mothers who were included. Excluded offspring had lower lung function, birthweight and IgE, and a lower prevalence of hayfever, eczema and allergic sensitisation, and a higher prevalence of wheezing and asthma than those who were included, although differences were small.

Table 1 shows the distribution of maternal and offspring characteristics, including respiratory and allergic outcomes, according to the quintiles of first haemoglobin measurement in pregnancy. First haemoglobin measurements were higher when taken earlier in gestation and were positively correlated with last haemoglobin measurements, and negatively with anaemia in late pregnancy. There was weak evidence for an association between haemoglobin measurements and childhood allergic sensitisation, but not with prevalence of other respiratory or allergic outcomes in the offspring. Mothers with the lowest haemoglobin concentrations were more likely to have twin pregnancies, to be non‐white, multiparous and older; they also had a lower BMI and a lower prevalence of asthma. Associations with the last haemoglobin measurement in pregnancy were broadly similar, although mothers with the highest haemoglobin measurements were more likely to have twin pregnancies, to be older, to be better educated and to have lower birthweight babies (Table S2). Mean first haemoglobin concentrations were higher than mean last haemoglobin concentrations (prevalence of anaemia 4.8% and 31.1%, respectively).

**Table 1:** Characteristics of mothers and offspring who had information on at least one set of outcome variables (questionnaire‐based, asthma, allergic sensitisation, IgE or lung function) by quintiles of the mother's first pregnancy haemoglobin measurement (total N = 7,270)

	Total N (%)	Quintiles of first haemoglobin % or mean (SD)					*P* for difference
		1^st^(≤11.8 g/dL)	2^nd^ (11.9‐12.3 g/dL)	3^rd^(12.4‐12.7 g/dL)	4^th^(12.8‐13.2 g/dL)	5^th^(≥13.3 g/dL)	
Gestation at first haemoglobin (wk)	7270	10.8 (2.9)	10.3 (2.6)	10.1 (2.6)	9.9 (2.5)	9.6 (2.5)	<.001
Last maternal haemoglobin (g/dL)	7270	11.03 (0.91)	11.24 (0.82)	11.40 (0.82)	11.54 (0.88)	11.87 (0.93)	<.001
Gestation at last haemoglobin (wk)	7270	34.5 (3.1)	34.3 (3.0)	34.3 (3.1)	34.2 (3.1)	34.4 (3.2)	.13
Late pregnancy anaemia	7270						
No	5006	52.7	63.5	71.8	75.6	85.1	<.001
Yes	2264	47.3	36.5	28.2	24.4	14.9	
Hayfever	6189						
No	5639	90.3	91.2	90.8	91.1	92.3	
Yes	550	9.7	8.8	9.2	8.9	7.7	.53
Eczema	6204						
No	5177	83.8	83.8	83.1	82.5	83.9	
Yes	1027	16.2	16.2	16.9	17.5	16.1	.85
Wheezing	6219						
No	5551	89.3	88.6	89.0	88.7	90.7	
Yes	668	10.7	11.4	11.0	11.3	9.3	.48
Asthma	6163						
No	4895	80.0	77.3	81.2	79.3	79.8	
Yes	1268	20.0	22.7	18.8	20.7	20.2	.19
Allergic sensitisation	4956						
No	3908	78.6	75.9	80.5	80.2	79.7	
Yes	1048	21.4	24.1	19.5	19.8	20.3	.07
IgE (ku/L)^a^	3772	59.7 (20.9, 179.0)	55.5 (21.2, 210.0)	55.4 (21.4, 178.0)	60.3 (21.3, 189.0)	59.5 (18.2, 201.0)	.83
FEV_1_ SD score^b^	5076	0.02 (1.00)	0.01 (1.00)	0.00 (0.98)	0.03 (1.00)	−0.02 (0.99)	.97
FVC SD score^b^	5149	0.00 (1.00)	0.00 (1.00)	0.04 (0.99)	0.01 (0.99)	−0.01 (1.00)	.80
FEF_25‐75_ SD score^b^	5149	0.02 (0.98)	0.01 (0.97)	−0.04 (1.02)	0.01 (1.00)	0.00 (1.03)	.65
Gender	7270						
Male	3749	51.8	52.6	51.8	49.6	51.9	
Female	3521	48.2	47.4	48.2	50.4	48.1	.55
Multiple pregnancy	7270						
Singleton	7106	96.5	98.6	97.4	98.2	98.2	
Twin	164	3.5	1.4	2.6	1.8	1.8	.001
Maternal ethnicity	6960						
White	6829	96.8	98.1	98.5	98.6	98.8	
Non‐white	131	3.2	1.9	1.5	1.4	1.2	<.001
Maternal BMI (kg/m^2^)	6563	22.36 (3.48)	22.66 (3.41)	22.93 (3.66)	23.21 (3.74)	23.84 (4.48)	<.001
Maternal age (y)	7270	28.93 (4.63)	28.93 (4.49)	28.79 (4.58)	28.78 (4.61)	28.34 (4.64)	.003
Maternal parity	7014						
Nulliparous	3304	41.8	43.7	47.3	49.3	54.9	
Multiparous	3710	58.2	56.3	52.7	50.7	45.1	<.001
Maternal smoking in pregnancy	7069						
Never	5146	70.4	74.5	73.4	73.7	72.3	
1^st^ trimester	857	12.1	12.2	13.6	11.2	11.6	
Throughout	1066	17.5	13.3	13.0	15.1	16.1	.01
Maternal education	6991						
CSE/vocational	1720	25.1	23.6	23.6	24.8	25.8	
O level	2510	35.4	36.0	34.8	35.9	37.4	
A level	1739	25.1	26.4	24.8	23.2	24.6	
Degree	1022	14.3	14.1	16.7	16.1	12.1	.11
Maternal asthma	7002						
No	6242	91.3	90.2	88.2	87.8	87.6	
Yes	760	8.7	9.8	11.8	12.2	12.4	.003
Maternal eczema	7002						
No	5370	77.1	78.9	78.0	74.7	74.7	
Yes	1632	22.9	21.1	22.0	25.3	25.3	.02
Gestational age (wk)^a^	7270	40 (39, 41)	40 (39, 41)	40 (39, 41)	40 (39, 41)	40 (39, 41)	.92
Birthweight (g)	7213	3442.4 (536.3)	3456.0 (509.1)	3445.0 (527.6)	3446.3 (533.2)	3404.2 (557.1)	.10

a Median (interquartile range) is presented due to the skewed distributions of these variables.

b Lung function SD scores are standardised by age, sex and height.

**Figure 1:**
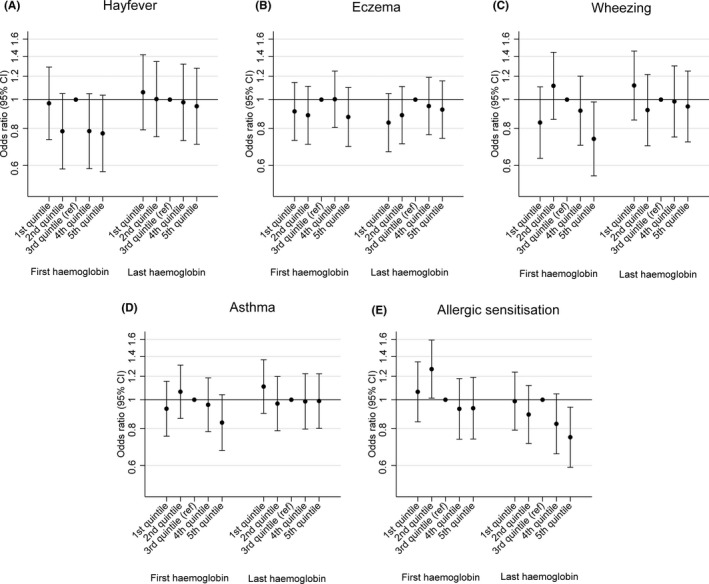
Associations of first and last maternal haemoglobin measurements in pregnancy with binary offspring outcomes. Adjusted for offspring sex, gestational age at the time of the haemoglobin measure, maternal asthma, maternal eczema, maternal pre‐pregnancy BMI, maternal age, maternal parity, maternal smoking during pregnancy, maternal education, multiple pregnancy and maternal ethnicity (Model 2)

Figure 1 and Table S3 and S4 show that neither first, nor last, haemoglobin measurements were associated with hayfever, eczema, wheezing or asthma in the offspring when haemoglobin was analysed as a continuous variable. Compared to mothers who were in the middle quintile for first haemoglobin measurement, those in the top quintile were less likely to have children who wheezed; this finding did not change on controlling for confounders or after further adjustment for gestational age and weight at birth (Table S3).

**Figure 2:**
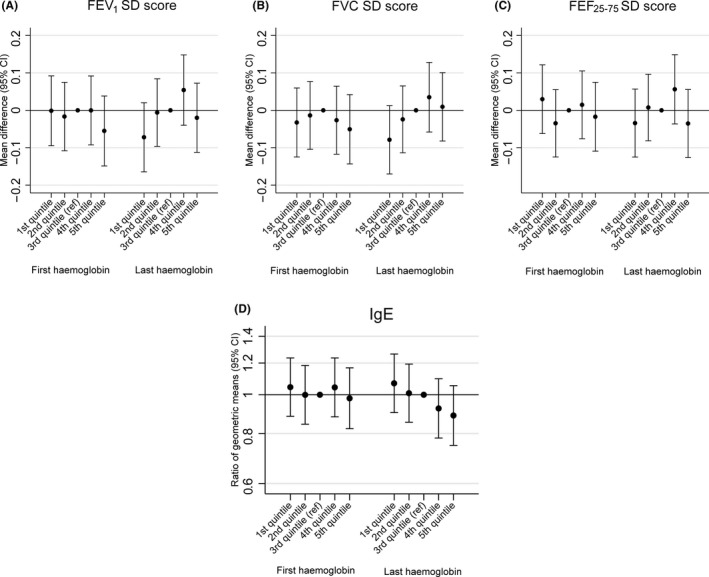
Associations of first and last maternal haemoglobin measurements in pregnancy with continuous offspring outcomes. Adjusted for offspring sex, gestational age at the time of the haemoglobin measure, maternal asthma, maternal eczema, maternal pre‐pregnancy BMI, maternal age, maternal parity, maternal smoking during pregnancy, maternal education, multiple pregnancy and maternal ethnicity (Model 2)

Figure 1 and Table 2 show that both first and last haemoglobin measurements were negatively associated with allergic sensitisation in the offspring after controlling for confounders. Children of mothers in the top quintile for last haemoglobin measurement were 25% less likely to be sensitized than those of mothers in the middle quintile. Last haemoglobin was also negatively associated with total IgE (Figure 2 and Table S5). These associations were unchanged on controlling additionally for gestational age and weight at birth. There was weak evidence for a positive association between last haemoglobin and offspring FVC, after controlling for confounders, which was not attenuated by controlling additionally for gestational age and weight at birth (Figure 2 and Table S6).

**Table 2:** Odds ratios (95% confidence interval) for allergic sensitisation associated with maternal first and last haemoglobin measurements in pregnancy (N = 4,235)^a^

	Model 1 Odds Ratio (95% CI)	Model 2 Odds Ratio (95% CI)	Model 3 Odds Ratio (95% CI)
**First haemoglobin**			
Per g/dL of haemoglobin	0.91 (0.83, 0.98)	0.91 (0.83, 0.99)	0.91 (0.83, 0.99)
*Quintiles of haemoglobin*			
1st quintile	1.06 (0.84, 1.34)	1.06 (0.84, 1.34)	1.06 (0.84, 1.34)
2nd quintile	1.24 (0.99, 1.55)	1.27 (1.01, 1.59)	1.28 (1.02, 1.61)
3rd quintile (reference)	1	1	1
4th quintile	0.94 (0.74, 1.18)	0.93 (0.74, 1.18)	0.93 (0.74, 1.18)
5th quintile	0.92 (0.73, 1.17)	0.94 (0.74, 1.19)	0.94 (0.74, 1.20)
**Last haemoglobin**			
Per g/dL of haemoglobin	0.92 (0.85, 1.00)	0.90 (0.83, 0.98)	0.90 (0.83, 0.98)
*Quintiles of haemoglobin*			
1st quintile	0.97 (0.78, 1.21)	0.99 (0.79, 1.24)	1.00 (0.80, 1.26)
2nd quintile	0.88 (0.71, 1.11)	0.89 (0.71, 1.12)	0.89 (0.71, 1.12)
3rd quintile (reference)	1	1	1
4th quintile	0.85 (0.67, 1.06)	0.83 (0.66, 1.05)	0.83 (0.66, 1.05)
5th quintile	0.78 (0.62, 0.98)	0.75 (0.59, 0.94)	0.76 (0.60, 0.96)

a Model 1 is adjusted for offspring sex and gestational age at the time of the haemoglobin measure; Model 2 is additionally adjusted for maternal asthma, maternal eczema, maternal pre‐pregnancy BMI, maternal age, maternal parity, maternal smoking during pregnancy, maternal education, multiple pregnancy and maternal ethnicity; Model 3 is additionally adjusted for gestational age at birth and birthweight.

In secondary analyses, we analysed associations with anaemia in late pregnancy. Anaemia was associated with a lower risk of eczema (Table S7), and there was weak evidence for a positive association with asthma (Table S8), but no association with allergic sensitisation or IgE (Table S9 and S10). Anaemia was associated with lower FVC (Table 3). We also tested whether the associations between maternal haemoglobin and each of the outcomes were modified by maternal asthma, but there was no evidence of interaction (all *P*‐values for interaction >.01; full stratified results available from authors on request).

**Table 3:** Mean difference (95% confidence interval) in lung function outcomes associated with maternal anaemia in late pregnancy (N = 4,343)^a^

	Model 1	Model 2	Model 3
	Mean difference (95% CI)	Mean difference (95% CI)	Mean difference (95% CI)
**FEV_1_ SD score** b			
*Late pregnancy anaemia*			
No	0	0	0
Yes	−0.05 (−0.11, 0.02)	−0.04 (−0.11, 0.02)	−0.06 (−0.12, 0.01)
**FVC SD score** b			
*Late pregnancy anaemia*			
No	0	0	0
Yes	−0.08 (−0.14, −0.01)	−0.07 (−0.13, −0.01)	−0.08 (−0.15, −0.02)
**FEF_25‐75_ SD score** b			
*Late pregnancy anaemia*			
No	0	0	0
Yes	0.01 (−0.05, 0.08)	0.01 (−0.06, 0.07)	0.00 (−0.06, 0.07)

a Model 1 is adjusted for offspring sex and gestational age at the time of the haemoglobin measure; Model 2 is additionally adjusted for maternal asthma, maternal eczema, maternal pre‐pregnancy BMI, maternal age, maternal parity, maternal smoking during pregnancy, maternal education, multiple pregnancy and maternal ethnicity; Model 3 is additionally adjusted for gestational age at birth and birthweight.

b Lung function SD scores are standardised by age, sex and height.

## 4 DISCUSSION

In this large population‐based birth cohort, we found that a lower maternal haemoglobin concentration in pregnancy was associated with an increased risk of allergic sensitisation and elevated IgE in the offspring. Lower haemoglobin and anaemia were also associated with lower FVC. A previous, much smaller, study of 597 mother‐child pairs by Triche et al in the USA reported that maternal anaemia measured prior to delivery was positively associated with risk of persistent wheezing and asthma at 6 years of age, but only amongst offspring of mothers with asthma.^15^ In ALSPAC, we found no association with asthma in the whole cohort when analysing haemoglobin as a continuous variable throughout pregnancy, although there was weak evidence for a positive association between anaemia in late pregnancy and asthma. However, we found no evidence for effect modification by maternal asthma. Given that our study was an order of magnitude larger, and hence had much greater statistical power, we think that the findings of the American study may have arisen by chance. That study did not investigate relations with allergic sensitisation or IgE. Furthermore, a large birth cohort study in the Netherlands also found no relation between maternal haemoglobin in pregnancy and offspring asthma.^16^ The only other study to examine the relation between maternal haemoglobin and childhood respiratory and allergic outcomes, including allergic sensitisation, analysed data from a very small subset of the Scottish SEATON cohort. However, statistical power was extremely limited by the small sample size (n = 157 mother‐child pairs), and because the exposure was analysed only as a dichotomous variable; no association was found between low maternal haemoglobin at delivery and any outcome.^11^ Whilst our analyses of anaemia and allergic sensitisation did not support the results for maternal haemoglobin analysed as a continuous variable, this may be because there was less statistical power when analysing a binary exposure and outcome.

One interpretation of our findings for maternal haemoglobin in pregnancy is that lower prenatal iron status increases the risk of allergic sensitisation and lower lung function in children of school age. However, to our knowledge, the only study to investigate this directly was the SEATON study, which analysed the relation between three different indicators of maternal iron status measured in early gestation and at delivery, and multiple respiratory and allergic outcomes in the offspring.^11^ Whilst one indicator of lower iron status measured at birth was positively associated with childhood allergic sensitisation, this may have been a chance finding, as the other indicators were not, and none of the indicators measured in early pregnancy were associated with allergic sensitisation. That study did, however, find some evidence for an association between lower prenatal iron status and lower lung function.

### 4.1 Mechanisms

If lower prenatal iron status does cause an increased risk of allergic sensitisation in childhood, we speculate that this might occur through persistence of Th2 immune responses postnatally. We would therefore expect iron status in late pregnancy to be more influential than in early pregnancy, and we found a clear reduction in allergic sensitisation amongst children of mothers in the top quintile for last haemoglobin which was not evident for first haemoglobin. However, whilst there are some animal and human data suggesting that iron deficiency may increase Th2 cytokine responses,^25^, ^26^, ^27^ other studies have not confirmed this.^28^, ^29^ Furthermore, interpretation of the direction of iron‐cytokine associations is complicated by the fact that Th1 and Th2 cytokines can influence iron metabolism.^30^, ^31^ Regarding the weak evidence linking lower maternal haemoglobin and anaemia to lower lung function, especially FVC, in the offspring, there is some evidence from animal experiments suggesting that an adequate supply of iron is needed for optimal lung development.^9^ Adjusting for birthweight and gestational age did not attenuate the associations of maternal haemoglobin with allergic sensitisation, IgE and lung function, suggesting that these associations were not mediated by impaired foetal growth overall. However, lower birth weight is a crude proxy measure of impaired foetal lung growth, which could still have mediated the relation between lower maternal haemoglobin and anaemia and lower childhood lung function.

### 4.2 Strengths and limitations

Aside from ALSPAC's size and population‐based prospective design, a strength of our study is that maternal haemoglobin was objectively measured as part of routine antenatal care and abstracted from the women's obstetric records, thus reducing the likelihood of selection or measurement bias. However, haemoglobin, which provides a measure of the functional use of iron, has two limitations as a biomarker of iron status in pregnancy. Firstly, whilst iron deficiency is the commonest cause of anaemia in pregnancy,^13^ mothers may be anaemic but not iron‐deficient, and conversely they may be mildly iron‐deficient and not anaemic.^13^ Secondly, haemoglobin concentrations vary as plasma volume increases during gestation, leading to haemodilution, with the greatest dilution and lowest haemoglobin concentrations occurring in the second trimester.^13^, ^32^ However, as the haemoglobin measurements used in our analyses were in the first and last trimesters, we think that haemodilution is unlikely to have influenced them to a great extent. Whilst we did not have measurements of other biomarkers of maternal iron status, such as plasma ferritin, plasma transferrin saturation, or serum soluble transferrin receptor and its ratio to ferritin,^33^ no single marker of iron metabolism is considered ideal for assessment of iron deficiency, as each has limitations in terms of sensitivity and specificity.^32^, ^34^, ^35^ A particular limitation of ferritin, which is an indirect indicator of total body iron stores, is that it is an acute phase reactant and will increase in the presence of inflammation.^36^ If, however, our findings for maternal haemoglobin do reflect effects of underlying iron deficiency, and given that misclassification of iron deficiency using haemoglobin as a biomarker is likely to be random with respect to childhood outcomes, then it is likely we will have underestimated effects of prenatal iron deficiency using maternal haemoglobin as a proxy marker.

Given the number of statistical tests carried out, we acknowledge that the main findings could have arisen by chance; it would therefore be of interest to see whether they can be replicated in other large birth cohorts. Whilst we cannot rule out the possibility that our main findings might be explained by uncontrolled or residual confounding, we controlled for a large number of potential confounders. We acknowledge that there may have been some misclassification of maternally‐reported outcomes, however, as misclassification of outcome is likely to be non‐differential with respect to the exposure of interest, this would be expected to bias effect estimates towards the null; in other words, the magnitude of associations might be underestimated. As with any longitudinal study, data were not complete on exposures, outcomes and confounders for the whole cohort. Therefore, we cannot rule out the possibility that exclusion of children without complete information might have biased our findings. However, we can see no reason why associations between maternal haemoglobin and the outcomes of interest would be different in those included and those excluded.

In conclusion, we have found some evidence to suggest that a lower maternal haemoglobin concentration in pregnancy is associated with an increased risk of allergic sensitisation, elevated IgE and lower FVC in the offspring. To test whether these findings reflect causal effects of prenatal iron deficiency, we plan to use a Mendelian randomisation approach, whereby maternal genotype will be used as a proxy for prenatal iron status. This approach has been used previously in ALSPAC to examine the association between maternal iron status and a different childhood outcome.^37^ Confirmation of a causal link could raise exciting possibilities for primary prevention through iron supplementation in pregnancy.

## CONFLICT OF INTEREST

The authors have no conflict of interest to declare.

## Supporting information


** **
