# Supplementary material for: Haemoglobin concentrations in pregnancy and respiratory and allergic outcomes in childhood: Birth cohort study
Source: Clin Exp Allergy. 2017 Oct 16;47(12):1615–24. doi: 10.1111/cea.13034 (PMC5725736; doi:10.1111/cea.13034)
Supplement: Supplementary file 1 [file CEA-47-1615-s001.docx]

Figure S1 Directed acyclic graph showing potential confounders and mediators of the associations between maternal haemoglobin in pregnancy and offspring respiratory and allergic outcomes

**Maternal haemoglobin in pregnancy**

**Confounders:**

maternal asthma, eczema, pre-pregnancy BMI, age, parity, smoking during pregnancy, education, multiple pregnancy, ethnicity

**Offspring outcomes:**

hayfever, eczema, wheeze, asthma, allergic sensitisation, IgE, lung function

**Mediators:**

gestational age at birth, birth weight

9 triplets and 4 quadruplets excluded

685 were not alive at 1 year

220 without consent or maternal medical records could not be located

649 with neither measure

1182 with first haemoglobin only

1729 with last haemoglobin only

14,541 recruited ALSPAC mothers

14,676 children from these pregnancies

13,978 singletons or twins alive at 1 year

13,758 with maternal data abstracted from medical records

10,198 with maternal haemoglobin measured before 18 weeks and after 28 weeks gestation

4,956 with skin prick test at 7.5 years from clinic

3,772 with IgE from blood sample at clinic at 7.5 years

6,174 with hayfever, eczema and wheezing data at 7.6 years from questionnaire

6,163 with asthma information at 7.6 years from questionnaire

5,076 with lung function measures at 8.5 years from clinic

4,235 with complete data on all maternal and offspring covariables

3,234 with complete data on all maternal and offspring covariables

5,335 with complete data on all maternal and offspring covariables

5,332 with complete data on all maternal and offspring covariables

4,343 with complete data on all maternal and offspring covariables

Figure S2 Participant flow

Table S1 Characteristics of mothers and offspring who were included in analyses for each of the outcomes and those who were excluded from all analyses due to incomplete data (total eligible = 10,198)

|  | | | **Complete data for hayfever, eczema and wheeze analysis**  **N=5,335** | | **Complete data for allergic sensitisation analysis**  **N=4,235** | | **Complete data for IgE analysis**  **N=3,234** | | **Complete data for asthma analysis**  **N=5,332** | | **Complete data for lung function analysis**  **N=4,343** | | **Excluded from all analyses due to incomplete data**  **N=4,061** | |
| --- | --- | --- | --- | --- | --- | --- | --- | --- | --- | --- | --- | --- | --- | --- |
|  |  |  | N | Mean (SD)/% | N | Mean (SD)/% | N | Mean (SD)/% | N | Mean (SD)/% | N | Mean (SD)/% | N | Mean (SD)/% |
| First maternal haemoglobin (g/dL) | | | 5335 | 12.49 (0.90) | 4235 | 12.49 (0.90) | 3234 | 12.46 (0.90) | 5332 | 12.48 (0.90) | 4343 | 12.49 (0.90) | 4061 | 12.42 (0.95) |
| Gestation at first haemoglobin (weeks) | | | 5335 | 10.2 (2.6) | 4235 | 10.2 (2.6) | 3234 | 10.3 (2.7) | 5332 | 10.2 (2.6) | 4343 | 10.2 (2.6) | 4061 | 10.2 (2.8) |
| Last maternal haemoglobin (g/dL) | | | 5335 | 11.41 (0.92) | 4235 | 11.40 (0.92) | 3234 | 11.39 (0.91) | 5332 | 11.41 (0.92) | 4343 | 11.41 (0.92) | 4061 | 11.26 (0.96) |
| Gestation at last haemoglobin (weeks) | | | 5335 | 34.3 (3.1) | 4235 | 34.4 (3.1) | 3234 | 34.3 (3.1) | 5332 | 34.3 (3.1) | 4343 | 34.3 (3.1) | 4061 | 34.3 (3.1) |
| Hayfever | | No | 4860 | 91.1 | 3347 | 90.8 | 2569 | 90.8 | 4823 | 91.0 | 3446 | 90.7 | 772 | 91.8 |
|  |  | Yes | 475 | 8.9 | 338 | 9.2 | 259 | 9.2 | 478 | 9.0 | 355 | 9.3 | 69 | 8.2 |
| Eczema | | No | 4436 | 83.1 | 3045 | 82.4 | 2366 | 83.5 | 4414 | 83.1 | 3132 | 82.3 | 721 | 85.4 |
|  |  | Yes | 899 | 16.9 | 649 | 17.6 | 467 | 16.5 | 899 | 16.9 | 674 | 17.7 | 123 | 14.6 |
| Wheezing | | No | 4771 | 89.4 | 3306 | 89.3 | 2537 | 89.4 | 4756 | 89.3 | 3396 | 89.0 | 750 | 88.5 |
|  |  | Yes | 564 | 10.6 | 396 | 10.7 | 301 | 10.6 | 569 | 10.7 | 419 | 11.0 | 97 | 11.5 |
| Asthma | | Yes | 4230 | 80.0 | 2944 | 80.1 | 2268 | 80.5 | 4259 | 79.9 | 3020 | 79.7 | 636 | 76.5 |
|  |  | No | 1058 | 20.0 | 733 | 19.9 | 549 | 19.5 | 1073 | 20.1 | 770 | 20.3 | 195 | 23.5 |
| Allergic sensitisation | | No | 2892 | 78.7 | 3328 | 78.6 | 2230 | 79.4 | 2883 | 78.4 | 2670 | 78.5 | 580 | 80.4 |
|  |  | Yes | 784 | 21.3 | 907 | 21.4 | 580 | 20.6 | 794 | 21.6 | 731 | 21.5 | 141 | 19.6 |
| IgE (ku/l)^*^ | | | 2823 | 59.3  (21.0, 193.0) | 2810 | 59.4  (20.8, 193.0) | 3234 | 59.1  (20.9, 199.0) | 2817 | 59.9  (21.2, 196.0) | 2621 | 60.2  (21.3, 201.0) | 538 | 55.9  (20.3, 166.0) |
| FEV_1_ SD score**^†^** | | | 3791 | 0.01 (1.00) | 3401 | 0.02 (0.99) | 2621 | 0.02 (1.00) | 3790 | 0.01 (1.00) | 4343 | 0.01 (1.00) | 733 | -0.03 (0.99) |
| FVC SD score**^†^** | | | 3845 | 0.01 (0.99) | 3451 | 0.01 (0.98) | 2662 | 0.02 (0.99) | 3845 | 0.01 (0.99) | 4343 | 0.03 (0.99) | 750 | -0.03 (1.00) |
| FEF_25-75_ SD score**^†^** | | | 3845 | 0.01 (1.01) | 3451 | 0.02 (1.00) | 2662 | 0.02 (1.01) | 3845 | 0.01 (1.01) | 4343 | -0.02 (0.98) | 750 | -0.02 (0.99) |
| Sex | | Male | 2758 | 51.7 | 2131 | 50.3 | 1678 | 51.9 | 2760 | 51.8 | 2198 | 50.6 | 2101 | 51.7 |
|  |  | Female | 2577 | 48.3 | 2104 | 49.7 | 1556 | 48.1 | 2572 | 48.2 | 2145 | 49.4 | 1960 | 48.3 |
| Multiple pregnancy | | Singleton | 5232 | 98.1 | 4139 | 97.7 | 3192 | 98.7 | 5230 | 98.1 | 4242 | 97.7 | 3958 | 97.5 |
|  |  | Twin | 103 | 1.9 | 96 | 2.3 | 42 | 1.3 | 102 | 1.9 | 101 | 2.3 | 103 | 2.5 |
| Maternal ethnicity | | White | 5249 | 98.4 | 4163 | 98.3 | 3189 | 98.6 | 5246 | 98.4 | 4280 | 98.5 | 2938 | 96.2 |
|  |  | Non-white | 86 | 1.6 | 72 | 1.7 | 45 | 1.4 | 86 | 1.6 | 63 | 1.5 | 115 | 3.8 |
| Maternal pre-pregnancy BMI (kg/m^2^) | | | 5335 | 22.87 (3.66) | 4235 | 22.92 (3.68) | 3234 | 22.88 (3.74) | 5332 | 22.87 (3.66) | 4343 | 22.98 (3.76) | 2490 | 23.03 (4.08) |
| Maternal age (years) | | | 5335 | 29.09 (4.43) | 4235 | 29.18 (4.45) | 3234 | 29.28 (4.46) | 5332 | 29.08 (4.42) | 4343 | 29.23 (4.39) | 4061 | 26.62 (5.01) |
| Parity | Nulliparous | | 2560 | 48.0 | 1983 | 46.8 | 1468 | 45.4 | 2562 | 48.0 | 2081 | 47.9 | 1486 | 43.7 |
|  | Multiparous | | 2775 | 52.0 | 2252 | 53.2 | 1766 | 54.6 | 2770 | 52.0 | 2262 | 52.1 | 1914 | 56.3 |
| Maternal smoking in pregnancy | Never | | 3988 | 74.8 | 3188 | 75.3 | 2455 | 75.9 | 3979 | 74.6 | 3277 | 75.5 | 1987 | 56.7 |
|  | 1^st^ trimester only | | 613 | 11.5 | 478 | 11.3 | 364 | 11.3 | 616 | 11.6 | 502 | 11.6 | 587 | 16.8 |
|  | Throughout | | 734 | 13.8 | 569 | 13.4 | 415 | 12.8 | 737 | 13.8 | 564 | 13.0 | 928 | 26.5 |
| Maternal education | CSE/  vocational | | 1173 | 22.0 | 872 | 20.6 | 646 | 20.0 | 1170 | 21.9 | 871 | 20.1 | 1329 | 42.6 |
|  | O level | | 1915 | 35.9 | 1518 | 35.8 | 1158 | 35.8 | 1918 | 36.0 | 1542 | 35.5 | 1052 | 33.7 |
|  | A level | | 1382 | 25.9 | 1137 | 26.8 | 879 | 27.2 | 1380 | 25.9 | 1187 | 27.3 | 516 | 16.5 |
|  | Degree | | 865 | 16.2 | 708 | 16.7 | 551 | 17.0 | 864 | 16.2 | 743 | 17.1 | 223 | 7.1 |
| Maternal asthma | No | | 4778 | 89.6 | 3761 | 88.8 | 2878 | 89.0 | 4776 | 89.6 | 3847 | 88.6 | 2768 | 87.9 |
|  | Yes | | 557 | 10.4 | 474 | 11.2 | 356 | 11.0 | 556 | 10.4 | 496 | 11.4 | 380 | 12.1 |
| Maternal eczema | No | | 4074 | 76.4 | 3206 | 75.7 | 2493 | 77.1 | 4068 | 76.3 | 3286 | 75.7 | 2514 | 79.9 |
|  | Yes | | 1261 | 23.6 | 1029 | 24.3 | 741 | 22.9 | 1264 | 23.7 | 1057 | 24.3 | 634 | 20.1 |
| Gestational age^*^ | | | 5335 | 40 (39, 41) | 4235 | 40 (39, 41) | 3234 | 40 (39, 41) | 5332 | 40 (39, 41) | 4343 | 40 (39, 41) | 4061 | 40 (39, 41) |
| Birth weight | | | 5335 | 3445.2 (520.1) | 4235 | 3448.7 (526.9) | 3234 | 3469.2 (515.6) | 5332 | 3447.3 (519.8) | 4343 | 3448.9 (525.3) | 3962 | 3383.4 (554.9) |

^*^ Median (interquartile range) are presented due to the skewed distributions of these variables

**^†^** Lung function SD scores are standardised by age, sex and height

Table S2 Characteristics of mothers and offspring who had information on at least one set of outcome variables (questionnaire-based, asthma, allergic sensitisation, IgE or lung function) by quintiles of the mother’s last pregnancy haemoglobin measurement (total N=7,270)

|  | | | | **Total N (%)** | **Quintiles of last haemoglobin % or mean (SD)** | | | | | ***P* for difference** |  |
| --- | --- | --- | --- | --- | --- | --- | --- | --- | --- | --- | --- |
|  |  |  |  |  | **1^st^**  **(<=10.7 g/dl)** | **2nd**  **(10.8-11.2 g/dl)** | **3^rd^**  **(11.3-11.6 g/dl)** | **4th**  **(11.7-12.1 g/dl)** | **5^th^**  **(>=12.2 g/dl)** |  |  |
| **First maternal haemoglobin (g/dL)** | | | | 7270 | 12.07 (0.89) | 12.30 (0.81) | 12.50 (0.81) | 12.63 (0.86) | 12.92 (0.94) | <0.001 |  |
| **Gestation at first haemoglobin (weeks)** | | | | 7270 | 10.1 (2.7) | 10.2 (2.6) | 10.2 (2.6) | 10.2 (2.6) | 10.2 (2.7) | 0.83 |  |
| **Gestation at last haemoglobin (weeks)** | | | | 7270 | 34.4 (2.9) | 34.1 (2.8) | 34.2 (3.0) | 34.2 (3.2) | 34.8 (3.4) | <0.001 |  |
| **Hayfever** | | | | 6189 |  |  |  |  |  |  |  |
|  | | | No | 5639 | 91.1 | 90.9 | 91.2 | 91.5 | 90.9 |  |  |
|  |  |  | Yes | 550 | 8.9 | 9.1 | 8.8 | 8.5 | 9.1 | 0.99 |  |
| **Eczema** | | | | 6204 |  |  |  |  |  |  |  |
|  | | | No | 5177 | 84.3 | 84.6 | 82.4 | 83.0 | 83.1 |  |  |
|  |  |  | Yes | 1027 | 15.7 | 15.4 | 17.6 | 17.0 | 16.9 | 0.53 |  |
| **Wheezing** | | | | 6219 |  |  |  |  |  |  |  |
|  | | | No | 5551 | 87.7 | 89.9 | 89.3 | 89.8 | 89.6 |  |  |
|  |  |  | Yes | 668 | 12.3 | 10.1 | 10.7 | 10.2 | 10.4 | 0.42 |  |
| **Asthma** | | |  | 6163 |  |  |  |  |  |  |  |
|  | | | No | 4895 | 77.8 | 79.9 | 79.6 | 80.7 | 79.2 |  |  |
|  | | | Yes | 1268 | 22.2 | 20.1 | 20.4 | 19.3 | 20.8 | 0.52 |  |
| **Allergic sensitisation** | | | | 4956 |  |  |  |  |  |  |  |
|  | | | No | 3908 | 78.1 | 79.4 | 76.9 | 79.8 | 80.6 |  |  |
|  |  |  | Yes | 1048 | 21.9 | 20.6 | 23.1 | 20.2 | 19.4 | 0.24 |  |
| **IgE (ku/l)^*^** | | | | 3772 | 58.3 (22.0, 218.0) | 58.1 (21.3, 188.0) | 60.8 (22.0, 200.0) | 56.3 (17.8, 177.0) | 56.1 (19.5, 166.0) | 0.12 |  |
| **FEV_1_ SD score^†^** | | | | 5076 | -0.05 (0.98) | 0.02 (1.01) | 0.01 (1.02) | 0.05 (1.00) | 0.00 (0.98) | 0.66 |  |
| **FVC SD score^†^** | | | | 5149 | -0.04 (0.98) | -0.01 (1.00) | 0.01 (1.03) | 0.03 (0.98) | 0.04 (0.98) | 0.38 |  |
| **FEF_25-75_ SD score^†^** | | | | 5149 | -0.03 (0.97) | 0.03 (1.00) | 0.00 (1.00) | 0.05 (1.05) | -0.04 (0.99) | 0.20 |  |
| **Gender** | | | | 7270 |  |  |  |  |  |  |  |
|  | | | Male | 3749 | 48.3 | 50.8 | 53.0 | 52.5 | 53.2 |  |  |
|  |  |  | Female | 3521 | 51.7 | 49.2 | 47.0 | 47.5 | 46.8 | 0.05 |  |
| **Multiple pregnancy** | | | | 7270 |  |  |  |  |  |  |  |
|  | | | Singleton | 7106 | 98.0 | 99.0 | 98.7 | 98.0 | 94.7 |  |  |
|  |  |  | Twin | 164 | 2.0 | 1.0 | 1.3 | 2.0 | 5.3 | <0.001 |  |
| **Maternal ethnicity** | | | | 6960 |  |  |  |  |  |  |  |
|  | | | White | 6829 | 96.7 | 98.2 | 98.6 | 98.2 | 98.8 |  |  |
|  |  |  | Non-white | 131 | 3.3 | 1.8 | 1.4 | 1.8 | 1.2 | 0.001 |  |
| **Maternal BMI (kg/m^2^)** | | | | 6563 | 22.68 (3.48) | 22.69 (3.65) | 23.00 (3.74) | 22.98 (3.76) | 23.54 (4.24) | <0.001 |  |
| **Maternal age (years)** | | | | 7270 | 28.31 (4.66) | 28.73 (4.72) | 28.80 (4.54) | 29.02 (4.45) | 29.00 (4.55) | <0.001 |  |
| **Maternal parity** | | | | 7014 |  |  |  |  |  |  |  |
|  | | | Nulliparous | 3304 | 38.0 | 42.2 | 47.5 | 49.4 | 59.1 |  |  |
|  | | | Multiparous | 3710 | 62.0 | 57.8 | 52.5 | 50.6 | 40.9 | <0.001 |  |
| **Maternal smoking in pregnancy** | | | | 7069 |  |  |  |  |  |  |  |
|  | | | Never | 5146 | 73.0 | 72.3 | 71.3 | 74.9 | 72.8 |  |  |
|  | | | 1^st^ trimester | 857 | 12.0 | 12.0 | 13.2 | 11.5 | 11.6 |  |  |
|  | | | Throughout | 1066 | 14.9 | 15.7 | 15.5 | 13.5 | 15.6 | 0.60 |  |
| **Maternal education** | | | | 6991 |  |  |  |  |  |  |  |
|  | | CSE/vocational | | 1720 | 28.3 | 23.6 | 24.8 | 22.2 | 24.1 |  |  |
|  | | O level | | 2510 | 35.7 | 38.9 | 36.0 | 34.5 | 34.2 |  |  |
|  | | A level | | 1739 | 23.5 | 23.0 | 25.3 | 26.8 | 26.0 |  |  |
|  | | Degree | | 1022 | 12.5 | 14.5 | 14.0 | 16.6 | 15.7 | 0.002 |  |
| **Maternal asthma** | | | | 7002 |  |  |  |  |  |  |  |
|  | No | | | 6242 | 89.8 | 89.2 | 88.8 | 89.7 | 88.4 |  |  |
|  | Yes | | | 760 | 10.2 | 10.8 | 11.2 | 10.3 | 11.6 | 0.74 |  |
| **Maternal eczema** | | | | 7002 |  |  |  |  |  |  |  |
|  | No | | | 5370 | 77.6 | 76.6 | 75.9 | 75.9 | 77.6 |  |  |
|  | Yes | | | 1632 | 22.4 | 23.4 | 24.1 | 24.1 | 22.4 | 0.71 |  |
| **Gestational age (weeks)^*^** | | | | 7270 | 40 (39, 41) | 40 (39, 41) | 40 (39, 41) | 40 (39, 41) | 40 (38, 41) | <0.001 |  |
| **Birth weight (g)** | | | | 7213 | 3492.0 (542.8) | 3508.6 (471.9) | 3475.8 (505.8) | 3418.4 (508.3) | 3287.6 (601.1) | <0.001 |  |

^*^ Median (interquartile range) are presented due to the skewed distributions of these variables

**^†^** Lung function SD scores are standardised by age, sex and height

**Table S3 Odds ratios (95% confidence interval) for hayfever, eczema and wheezing associated with maternal first and last haemoglobin measurements in pregnancy (N=5,335)***

|  | **Model 1**  **Odds Ratio (95% CI)** | **Model 2**  **Odds Ratio (95% CI)** | **Model 3**  **Odds Ratio (95% CI)** |
| --- | --- | --- | --- |
| **First haemoglobin** | | | |
| **Hayfever**  Per g/dL of haemoglobin  Quintiles of haemoglobin:  1^st^ quintile  2^nd^ quintile  3^rd^ quintile (reference)  4^th^ quintile  5^th^ quintile | 0.94 (0.85, 1.05)  0.95 (0.72, 1.26)  0.76 (0.57, 1.02)  1  0.79 (0.59, 1.05)  0.79 (0.59, 1.05) | 0.92 (0.83, 1.03)  0.97 (0.73, 1.29)  0.78 (0.58, 1.05)  1  0.78 (0.58, 1.05)  0.77 (0.57, 1.03) | 0.92 (0.83, 1.03)  0.97 (0.73, 1.29)  0.79 (0.59, 1.05)  1  0.78 (0.59, 1.05)  0.77 (0.57, 1.04) |
| **Eczema**  Per g/dL of haemoglobin  Quintiles of haemoglobin:  1^st^ quintile  2^nd^ quintile  3^rd^ quintile (reference)  4^th^ quintile  5^th^ quintile | 1.00 (0.92, 1.08)  0.93 (0.74, 1.16)  0.87 (0.69, 1.08)  1  1.02 (0.82, 1.26)  0.87 (0.69, 1.09) | 1.00 (0.92, 1.09)  0.91 (0.73, 1.14)  0.89 (0.71, 1.11)  1  1.00 (0.81, 1.25)  0.87 (0.69, 1.10) | 1.00 (0.92, 1.09)  0.91 (0.73, 1.14)  0.89 (0.71, 1.12)  1  1.00 (0.80, 1.25)  0.88 (0.70, 1.11) |
| **Wheezing**  Per g/dL of haemoglobin  Quintiles of haemoglobin:  1^st^ quintile  2^nd^ quintile  3^rd^ quintile (reference)  4^th^ quintile  5^th^ quintile | 1.00 (0.90, 1.10)  0.83 (0.63, 1.09)  1.08 (0.83, 1.40)  1  0.93 (0.72, 1.22)  0.75 (0.56, 0.99) | 0.98 (0.89, 1.09)  0.84 (0.63, 1.10)  1.11 (0.86, 1.44)  1  0.92 (0.70, 1.20)  0.74 (0.55, 0.98) | 0.99 (0.88, 1.08)  0.84 (0.64, 1.11)  1.11 (0.86, 1.44)  1  0.92 (0.70, 1.20)  0.73 (0.55, 0.98) |
| **Last haemoglobin** | | | |
| **Hayfever**  Per g/dL of haemoglobin  Quintiles of haemoglobin:  1^st^ quintile  2^nd^ quintile  3^rd^ quintile (reference)  4^th^ quintile  5^th^ quintile | 0.98 (0.88, 1.09)  1.04 (0.78, 1.39)  0.98 (0.73, 1.31)  1  0.98 (0.73, 1.32)  0.98 (0.73, 1.31) | 0.96 (0.87, 1.07)  1.06 (0.79, 1.42)  1.00 (0.75, 1.35)  1  0.98 (0.73, 1.32)  0.95 (0.71, 1.28) | 0.96 (0.86, 1.07)  1.07 (0.80, 1.43)  1.01 (0.75, 1.35)  1  0.98 (0.73, 1.32)  0.96 (0.71, 1.29) |
| **Eczema**  Per g/dL of haemoglobin  Quintiles of haemoglobin:  1^st^ quintile  2^nd^ quintile  3^rd^ quintile (reference)  4^th^ quintile  5^th^ quintile | 1.03 (0.96, 1.12)  0.82 (0.66, 1.03)  0.88 (0.70, 1.09)  1  0.96 (0.77, 1.20)  0.93 (0.75, 1.16) | 1.02 (0.95, 1.11)  0.84 (0.67, 1.05)  0.89 (0.71, 1.11)  1  0.95 (0.76, 1.19)  0.93 (0.74, 1.16) | 1.03 (0.95, 1.12)  0.84 (0.67, 1.06)  0.89 (0.71, 1.11)  1  0.96 (0.77, 1.20)  0.96 (0.76, 1.20) |
| **Wheezing**  Per g/dL of haemoglobin  Quintiles of haemoglobin:  1^st^ quintile  2^nd^ quintile  3^rd^ quintile (reference)  4^th^ quintile  5^th^ quintile | 0.97 (0.88, 1.06)  1.10 (0.84, 1.43)  0.91 (0.69, 1.20)  1  0.99 (0.76, 1.31)  0.98 (0.74, 1.28) | 0.95 (0.86, 1.05)  1.12 (0.85, 1.46)  0.92 (0.70, 1.22)  1  0.99 (0.75, 1.30)  0.95 (0.72, 1.25) | 0.94 (0.86, 1.04)  1.11 (0.85, 1.45)  0.92 (0.70, 1.22)  1  0.98 (0.74, 1.29)  0.92 (0.69, 1.21) |

*****Model 1 is adjusted for offspring sex and gestational age at the time of the haemoglobin measure;

Model 2 is additionally adjusted for maternal asthma, maternal eczema, maternal pre-pregnancy BMI, maternal age, maternal parity, maternal smoking during pregnancy, maternal education, multiple pregnancy and maternal ethnicity;

Model 3 is additionally adjusted for gestational age at birth and birth weight

**Table S4 Odds ratios (95% confidence interval) for asthma associated with maternal first and last haemoglobin measurements in pregnancy (N=5,332)***

|  | **Model 1**  **Odds Ratio (95% CI)** | **Model 2**  **Odds Ratio (95% CI)** | **Model 3**  **Odds Ratio (95% CI)** |
| --- | --- | --- | --- |
| **First haemoglobin** | | | |
| Per g/dL of haemoglobin  Quintiles of haemoglobin:  1^st^ quintile  2^nd^ quintile  3^rd^ quintile (reference)  4^th^ quintile  5^th^ quintile | 1.00 (0.93, 1.08)  0.91 (0.74, 1.12)  1.02 (0.83, 1.25)  1  0.98 (0.80, 1.21)  0.88 (0.71, 1.08) | 0.96 (0.89, 1.04)  0.93 (0.75, 1.15)  1.06 (0.87, 1.31)  1  0.96 (0.78, 1.19)  0.84 (0.67, 1.04) | 0.96 (0.89, 1.04)  0.93 (0.75, 1.16)  1.06 (0.86, 1.31)  1  0.96 (0.78, 1.19)  0.83 (0.67, 1.04) |
| **Last haemoglobin** | | | |
| Per g/dL of haemoglobin  Quintiles of haemoglobin:  1^st^ quintile  2^nd^ quintile  3^rd^ quintile (reference)  4^th^ quintile  5^th^ quintile | 0.98 (0.91, 1.05)  1.09 (0.89, 1.34)  0.95 (0.78, 1.18)  1  0.96 (0.78, 1.19)  1.01 (0.82, 1.24) | 0.97 (0.90, 1.04)  1.11 (0.90, 1.36)  0.97 (0.79, 1.20)  1  0.99 (0.80, 1.22)  0.99 (0.80, 1.22) | 0.96 (0.89, 1.04)  1.10 (0.90, 1.36)  0.97 (0.79, 1.20)  1  0.98 (0.79, 1.22)  0.97 (0.78, 1.20) |

*****Model 1 is adjusted for offspring sex and gestational age at the time of the haemoglobin measure;

Model 2 is additionally adjusted for maternal asthma, maternal eczema, maternal pre-pregnancy BMI, maternal age, maternal parity, maternal smoking during pregnancy, maternal education, multiple pregnancy and maternal ethnicity;

Model 3 is additionally adjusted for gestational age at birth and birth weight

**Table S5 Ratio of geometric means (95% confidence interval) of IgE associated with maternal first and last haemoglobin measurements in pregnancy (N=3,234)***

|  | **Model 1**  **Ratio of Geometric Means (95% CI)** | **Model 2**  **Ratio of Geometric Means (95% CI)** | **Model 3**  **Ratio of Geometric Means (95% CI)** |
| --- | --- | --- | --- |
| **First haemoglobin** | | | |
| Per g/dL of haemoglobin  Quintiles of haemoglobin:  1^st^ quintile  2^nd^ quintile  3^rd^ quintile (reference)  4^th^ quintile  5^th^ quintile | 0.98 (0.93, 1.05)  1.04 (0.88, 1.22)  0.99 (0.83, 1.17)  1  1.04 (0.87, 1.23)  0.96 (0.81, 1.15) | 0.98 (0.93, 1.05)  1.04 (0.88, 1.24)  1.00 (0.84, 1.18)  1  1.04 (0.88, 1.24)  0.98 (0.82, 1.17) | 0.99 (0.93, 1.05)  1.04 (0.88, 1.23)  1.00 (0.85, 1.18)  1  1.04 (0.88, 1.24)  0.98 (0.83, 1.17) |
| **Last haemoglobin** | | | |
| Per g/dL of haemoglobin  Quintiles of haemoglobin:  1^st^ quintile  2^nd^ quintile  3^rd^ quintile (reference)  4^th^ quintile  5^th^ quintile | 0.92 (0.87, 0.98)  1.09 (0.92, 1.29)  1.02 (0.87, 1.21)  1  0.93 (0.79, 1.11)  0.88 (0.74, 1.04) | 0.94 (0.88, 0.99)  1.07 (0.90, 1.26)  1.01 (0.85, 1.19)  1  0.92 (0.78, 1.10)  0.89 (0.75, 1.05) | 0.94 (0.88, 1.00)  1.08 (0.91, 1.27)  1.01 (0.85, 1.19)  1  0.93 (0.78, 1.10)  0.90 (0.76, 1.08) |

*****Model 1 is adjusted for offspring sex and gestational age at the time of the haemoglobin measure;

Model 2 is additionally adjusted for maternal asthma, maternal eczema, maternal pre-pregnancy BMI, maternal age, maternal parity, maternal smoking during pregnancy, maternal education, multiple pregnancy and maternal ethnicity;

Model 3 is additionally adjusted for gestational age at birth and birth weight

**Table S6 Mean difference (95% confidence interval) in lung function outcomes associated with maternal first and last haemoglobin measurements in pregnancy (N=4,343)***

|  | **Model 1**  **Mean difference**  **(95% CI)** | **Model 2**  **Mean difference**  **(95% CI)** | **Model 3**  **Mean difference**  **(95% CI)** |
| --- | --- | --- | --- |
| **First haemoglobin** | | | |
| **FEV_1_ SD score^†^**  Per g/dL of haemoglobin  Quintiles of haemoglobin:  1^st^ quintile  2^nd^ quintile  3^rd^ quintile (reference)  4^th^ quintile  5^th^ quintile | -0.01 (-0.05, 0.02)  -0.01 (-0.10, 0.09)  -0.01 (-0.10, 0.08)  0  0.00 (-0.09, 0.09)  -0.05 (-0.14, 0.04) | -0.02 (-0.05, 0.02)  0.00 (-0.09, 0.09)  -0.02 (-0.11, 0.07)  0  0.00 (-0.09, 0.09)  -0.06 (-0.15, 0.04) | -0.01 (-0.05, 0.02)  -0.01 (-0.10, 0.09)  -0.02 (-0.11, 0.07)  0  0.00 (-0.10, 0.09)  -0.05 (-0.15, 0.04) |
| **FVC SD score^†^**  Per g/dL of haemoglobin  Quintiles of haemoglobin:  1^st^ quintile  2^nd^ quintile  3^rd^ quintile (reference)  4^th^ quintile  5^th^ quintile | 0.00 (-0.03, 0.04)  -0.04 (-0.13, 0.05)  -0.01 (-0.10, 0.08)  0  -0.02 (-0.11, 0.07)  -0.04 (-0.13, 0.05) | 0.00 (-0.04, 0.03)  -0.03 (-0.12, 0.06)  -0.01 (-0.10, 0.08)  0  -0.03 (-0.12, 0.06)  -0.05 (-0.14, 0.04) | 0.00 (-0.03, 0.03)  -0.04 (-0.13, 0.05)  -0.02 (-0.11, 0.07)  0  -0.03 (-0.12, 0.06)  -0.05 (-0.14, 0.04) |
| **FEF_25-75_  SD score^†^**  Per g/dL of haemoglobin  Quintiles of haemoglobin:  1^st^ quintile  2^nd^ quintile  3^rd^ quintile (reference)  4^th^ quintile  5^th^ quintile | -0.02 (-0.05, 0.01)  0.03 (-0.06, 0.12)  -0.03 (-0.12, 0.06)  0  0.01 (-0.08, 0.10)  -0.02 (-0.11, 0.07) | -0.02 (-0.05, 0.01)  0.03 (-0.06, 0.12)  -0.03 (-0.12, 0.06)  0  0.01 (-0.08, 0.11)  -0.02 (-0.11, 0.07) | -0.02 (-0.05, 0.02)  0.03 (-0.07, 0.12)  -0.03 (-0.12, 0.06)  0  0.01 (-0.08, 0.10)  -0.02 (-0.11, 0.08) |
| **Last haemoglobin** | | | |
| **FEV_1_ SD score^†^**  Per g/dL of haemoglobin  Quintiles of haemoglobin:  1^st^ quintile  2^nd^ quintile  3^rd^ quintile (reference)  4^th^ quintile  5^th^ quintile | 0.02 (-0.02, 0.05)  -0.08 (-0.17, 0.02)  -0.01 (-0.10, 0.08)  0  0.06 (-0.04, 0.15)  -0.03 (-0.12, 0.06) | 0.02 (-0.02, 0.05)  -0.07 (-0.16, 0.02)  -0.01 (-0.10, 0.08)  0  0.05 (-0.04, 0.15)  -0.02 (-0.11, 0.07) | 0.03 (-0.00, 0.06)  -0.08 (-0.17, 0.01)  -0.01 (-0.10, 0.08)  0  0.06 (-0.03, 0.16)  0.01 (-0.09, 0.10) |
| **FVC SD score^†^**  Per g/dL of haemoglobin  Quintiles of haemoglobin:  1^st^ quintile  2^nd^ quintile  3^rd^ quintile (reference)  4^th^ quintile  5^th^ quintile | 0.03 (-0.00, 0.06)  -0.09 (-0.18, 0.00)  -0.02 (-0.11, 0.06)  0  0.03 (-0.06, 0.13)  0.01 (-0.08, 0.10) | 0.03 (-0.00, 0.06)  -0.08 (-0.17, 0.01)  -0.02 (-0.11, 0.07)  0  0.03 (-0.06, 0.13)  0.01 (-0.08, 0.10) | 0.04 (0.01, 0.07)  -0.09 (-0.18, 0.00)  -0.03 (-0.12, 0.06)  0  0.04 (-0.05, 0.14)  0.03 (-0.06, 0.12) |
| **FEF_25-75_ SD score^†^**  Per g/dL of haemoglobin  Quintiles of haemoglobin:  1^st^ quintile  2^nd^ quintile  3^rd^ quintile (reference)  4^th^ quintile  5^th^ quintile | 0.00 (-0.04, 0.03)  -0.03 (-0.12, 0.06)  0.01 (-0.08, 0.10)  0  0.06 (-0.03, 0.16)  -0.05 (-0.14, 0.04) | 0.00 (-0.03, 0.03)  -0.03 (-0.13, 0.06)  0.01 (-0.08, 0.10)  0  0.06 (-0.04, 0.15)  -0.04 (-0.13, 0.06) | 0.01 (-0.02, 0.04)  -0.04 (-0.13, 0.06)  0.01 (-0.08, 0.09)  0  0.06 (-0.03, 0.15)  -0.02 (-0.11, 0.07) |

*****Model 1 is adjusted for offspring sex and gestational age at the time of the haemoglobin measure;

Model 2 is additionally adjusted for maternal asthma, maternal eczema, maternal pre-pregnancy BMI, maternal age, maternal parity, maternal smoking during pregnancy, maternal education, multiple pregnancy and maternal ethnicity;

Model 3 is additionally adjusted for gestational age at birth and birth weight

**^†^** Lung function SD scores are standardised by age, sex and height

Table S7: Odds ratios (95% confidence interval) for hayfever, eczema and wheezing associated with maternal anaemia in late pregnancy (N=5,335)*

|  | **Model 1**  **Odds Ratio (95% CI)** | **Model 2**  **Odds Ratio (95% CI)** | **Model 3**  **Odds Ratio (95% CI)** |
| --- | --- | --- | --- |
| **Hayfever**  Late pregnancy anaemia:  No  Yes | 1  1.02 (0.83, 1.25) | 1  1.05 (0.85, 1.29) | 1  1.05 (0.85, 1.30) |
| **Eczema**  Late pregnancy anaemia:  No  Yes | 1  0.81 (0.69, 0.95) | 1  0.81 (0.69, 0.95) | 1  0.81 (0.68, 0.95) |
| **Wheezing**  Late pregnancy anaemia:  No  Yes | 1  1.03 (0.85, 1.24) | 1  1.05 (0.86, 1.27) | 1  1.05 (0.87, 1.28) |

*****Model 1 is adjusted for offspring sex and gestational age at the time of the haemoglobin measure;

Model 2 is additionally adjusted for maternal asthma, maternal eczema, maternal pre-pregnancy BMI, maternal age, maternal parity, maternal smoking during pregnancy, maternal education, multiple pregnancy and maternal ethnicity;

Model 3 is additionally adjusted for gestational age at birth and birth weight

|  | **Model 1**  **Odds Ratio (95% CI)** | **Model 2**  **Odds Ratio (95% CI)** | **Model 3**  **Odds Ratio (95% CI)** |
| --- | --- | --- | --- |
| **Asthma**  Late pregnancy anaemia:  No  Yes | 1  1.12 (0.97, 1.29) | 1  1.14 (0.98, 1.32) | 1  1.14 (0.98, 1.32) |

Table S8: Odds ratios (95% confidence interval) for asthma associated with maternal anaemia in late pregnancy (N=5,332)*

*****Model 1 is adjusted for offspring sex and gestational age at the time of the haemoglobin measure;

Model 2 is additionally adjusted for maternal asthma, maternal eczema, maternal pre-pregnancy BMI, maternal age, maternal parity, maternal smoking during pregnancy, maternal education, multiple pregnancy and maternal ethnicity;

Model 3 is additionally adjusted for gestational age at birth and birth weight

|  | **Model 1**  **Odds Ratio (95% CI)** | **Model 2**  **Odds Ratio (95% CI)** | **Model 3**  **Odds Ratio (95% CI)** |
| --- | --- | --- | --- |
| **Allergic sensitisation**  Late pregnancy anaemia:  No  Yes | 1  1.05 (0.89, 1.23) | 1  1.08 (0.92, 1.27) | 1  1.08 (0.92, 1.28) |

Table S9: Odds ratios (95% confidence interval) for allergic sensitisation associated with maternal anaemia in late pregnancy (N=4,235)*

*Model 1 is adjusted for offspring sex and gestational age at the time of the haemoglobin measure;

Model 2 is additionally adjusted for maternal asthma, maternal eczema, maternal pre-pregnancy BMI, maternal age, maternal parity, maternal smoking during pregnancy, maternal education, multiple pregnancy and maternal ethnicity;

Model 3 is additionally adjusted for gestational age at birth and birth weight

Table S10: Ratio of geometric means (95% confidence interval) of IgE associated with maternal first and last haemoglobin measurements in pregnancy (N=3,234)*

|  | **Model 1**  **Ratio of geometric means (95% CI)** | **Model 2**  **Ratio of geometric means (95% CI)** | **Model 3**  **Ratio of geometric means (95% CI)** |
| --- | --- | --- | --- |
| **IgE**  Late pregnancy anaemia:  No  Yes | 1  1.09 (0.97, 1.23) | 1  1.07 (0.95, 1.21) | 1  1.07 (0.95, 1.21) |

*****Model 1 is adjusted for offspring sex and gestational age at the time of the haemoglobin measure;

Model 2 is additionally adjusted for maternal asthma, maternal eczema, maternal pre-pregnancy BMI, maternal age, maternal parity, maternal smoking during pregnancy, maternal education, multiple pregnancy and maternal ethnicity;

Model 3 is additionally adjusted for gestational age at birth and birth weight
